# Effect of Electromagnetic Field on Proliferation and Migration of Fibroblasts and Keratinocytes: Implications in Wound Healing and Regeneration

**DOI:** 10.26502/jbb.2642-91280162

**Published:** 2024-09-04

**Authors:** Marija Stojanovic, Vikrant Rai, Devendra K. Agrawal

**Affiliations:** 1 Department of Translational Research, Western University of Health Sciences, Pomona, California 91766, USA; 2 Institute of Medical Physiology “Richard Burian”, Faculty of Medicine, University of Belgrade, 11000 Belgrade, Serbia

**Keywords:** Cell Migration, Cell Proliferation, Electromagnetic field, Fibroblasts, Keratinocytes, Microglia, Neurons, Traumatic Brain Injury, Wound healing

## Abstract

Proliferation and migration of fibroblasts, keratinocytes, and endothelial cells are key events in the physiological process of wound healing. This process includes different but overlapping stages: hemostasis, inflammatory phase, the proliferative phase, and the remodeling phase. Traumatic brain injury (TBI) is defined as a mechanical insult to the brain from external mechanical force (primary injury), usually followed by the secondary injury including edema, inflammation, excitotoxicity, oxidative stress, or mitochondrial dysfunction. The process of tissue repair following TBI is based on the neuronal-glial interactions, where phagocytosis by microglia plays a crucial role. Low-frequency electromagnetic field (LF-EMF) has been shown to enhance tissue repair after TBI, however, there are limited studies investigating the effects of LF-EMF on the proliferation and migration of keratinocytes, fibroblasts, VSMCs, and endothelial cells in the context of wound healing and on neuronal cells and microglia in relation to healing after TBI. Better understanding of the effects of LF-EMF on the proliferation, migration, and differentiation of these cells is important to enhance tissue healing after injury. This review article comprehensively discussed the effect of EMF/LF-EMF on these cells. Results published by different authors are hardly comparable due to different methodological approach and experimental settings. EMF promotes migration and proliferation of fibroblasts, keratinocytes and endothelial cells (EC), and thus could improve wound healing. The pilot study preformed on a large animal model of TBI suggests anti-inflammatory effects of EMF stimulation following TBI. Therefore, EMF is recognized as a potential therapeutic option to accelerate the wound healing and improve cellular recovery and function after TBI. Nonetheless, future studies are needed to define the optimal parameters of EMF stimulation in terms of frequency or duration of exposure.

## Introduction

The process of tissue repair after trauma involves different cellular responses depending on the trauma and the type of damaged tissue. In general, wound healing is considered a protective mechanism of the body that restores tissue functions [1]. In a period of two to ten days after a skin injury, the proliferation and migration of several types of cells (fibroblasts, keratinocytes, endothelial cells) ensue, leading to the formation of a new tissue at the site of the injury [2, 3]. However, alteration in the normal wound healing and regeneration after injury leads to delayed wound healing or chronicity of the wound which is mainly due to arrest of wound in the inflammatory phase without progressing to proliferative phase. Persistent wound inflammation and decreased cellular proliferation, angiogenesis, and extracellular matrix (ECM) remodeling contribute to wound chronicity and impaired healing [4, 5]. Impaired wound healing is a socioeconomic burden with economical and psychological consequences. Thus, there is a need of delineating better strategies to promote wound healing and tissue repair. Recent literature has reported different effects of electromagnetic field (EMF) on various cells involved in the process of tissue repair. The effects of EMF on all phases of wound healing, including the hemostasis phase, inflammatory phase, proliferative phase, and the remodeling phase, were examined [6] and the results support the effectiveness of low frequency electromagnetic field (LF-EMF) in modulating inflammation, ECM remodeling, neo-angiogenesis, cell proliferation, and wound healing. EMF stimulation achieves biological effects by acting on various cells involved in the process of tissue repair following the injury, which indicate the importance of potential therapeutic application of this methodology in a clinical setting. Nevertheless, available research results are sometimes hardly comparable. These data are quite heterogeneous due to different methodological approach (study design) in terms of stimulation parameters that include different frequency range, amplitude, exposure time or waveform. Additionally, factors that also influence the final effect of EMF stimulation are the type of the cell as well as individual cell responsiveness [7–9]. Proliferation, migration, and recruitment of the keratinocytes, fibroblasts, vascular smooth muscle cells (VSMCs), and endothelial cells in wound healing while neuronal cells and microglia in brain tissue injury play a critical role. Various factors regulate the heterogeneity and phenotypic change of fibroblasts, angiogenesis, and inflammation during wound healing [10, 11] and LF-EMF plays a beneficial role in healing after TBI [12, 13]. However, studies investigating the effects of LF-EMF on the proliferation and migration of keratinocytes, fibroblasts, VSMCs, and endothelial cells in the context of wound healing and on neuronal cells and microglia in relation to healing after TBI are limited. Additionally, the change in the phenotypic properties (proliferation and migration) and phenotype after LF-EMF application has also not been investigated. Thus, there is a need to better understand the effects of EMF/LF-EMF on the proliferation and migration of cells involved in wound healing and tissue repair. This article provides a comprehensive and critical discussion on this process.

### Normal wound healing

**Figure 1:**
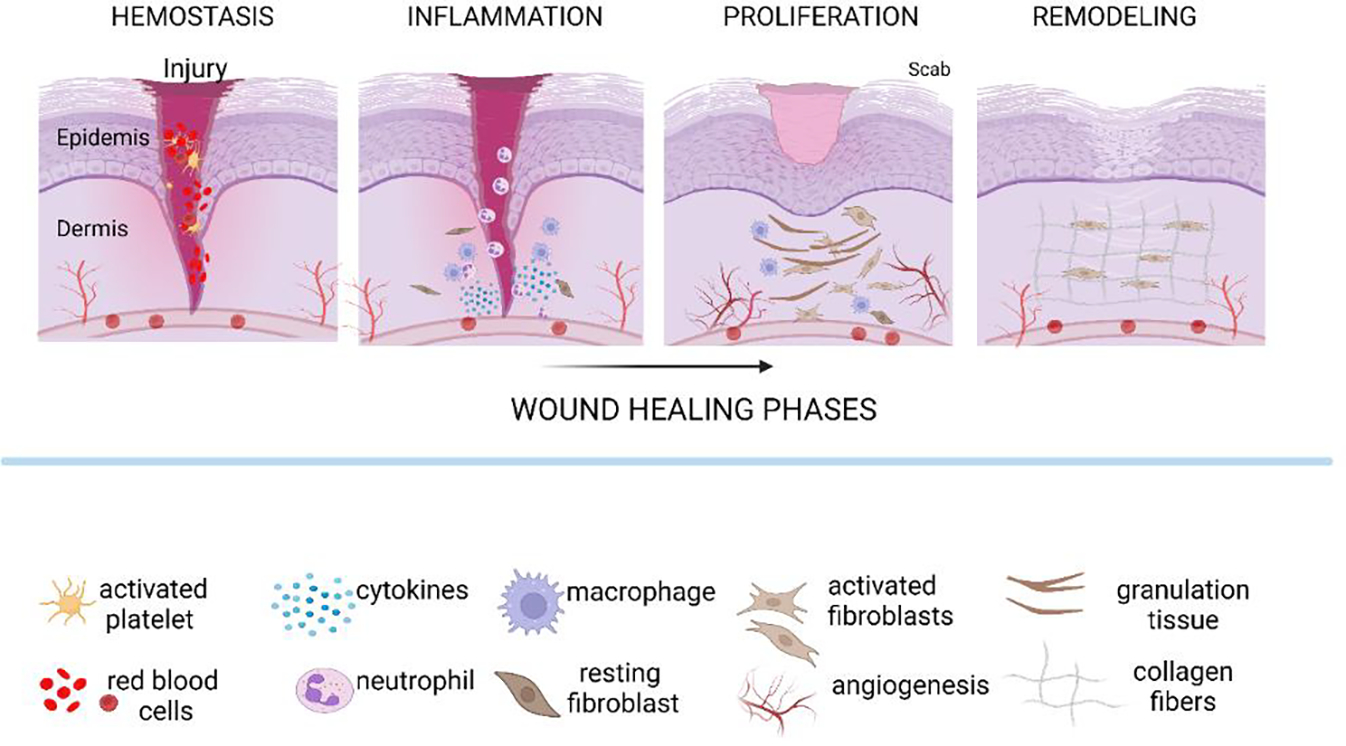
Physiology of wound healing process. Wound healing dynamics typically includes four continuous and overlapping phases: hemostasis, inflammatory phase, proliferation phase and remodeling phase. Immediately after a tissue injury in the hemostasis phase activated platelets start the reparation process by growth factors production. Inflammatory phase is divided into early (predominant neutrophil action) and late phase (predominant macrophage action) aimed to protect the tissue from foreign particles. Extracellular matrix deposition and angiogenesis contribute to granulation tissue formation in the proliferative phase. Definitive healing of the wound occurs in the remodeling phase, through complete epithelization and scar formation.

The physiological process of wound healing typically includes several phases: hemostasis, inflammatory phase, the proliferative phase, and the remodeling phase (Figure 1). The initial event for the hemostasis phase includes the process of platelet activation initiated after tissue injury. In this way, the arriving platelets adhere to the subendothelial collagen, their aggregation occurs, which leads to degranulation and the release of chemokines and growth factors, which in final leads to definitive hemostasis [6]. Tissue macrophages and monocytes in the blood contribute to successful wound healing because, in addition to their phagocytic role, they produce molecules that mediate the activation of fibroblasts, endothelial cells and keratinocytes [14]. The proliferative phase includes accumulation of fibroblasts, keratinocytes and endothelial cells and usually lasts for days and weeks. It is precisely this phase in which tissue renewal begins. The first fibroblasts inhabit the wound area approximately two to five days after the injury. A week or two after the initial injury, the number of fibroblasts in this local area reaches its maximum [15]. Activated fibroblasts synthesize ECM components, especially collagen. Adequate oxygen supply is necessary for appropriate collagen synthesis. This is especially important for the synthesis of mature collagen. Additionally, fibroblasts participate in the synthesis of elastin and the organization of the ECM [16], the bed for angiogenesis [10, 11]. Fibroblast growth factor (FGF) derived from macrophages, mast cells or T lymphocytes is responsible for fibroblast recruitment in tissue [17]. Additional factors released in the tissue like matrix metalloproteinase-14 (MMP-14) are responsible for the improvement of angiogenesis and collagen formation [18, 19]. Platelet-derived growth factor (PDGF) is another mediator released by the platelets, macrophages and fibroblasts leading to fibroblast recruitment and myofibroblast stimulation. Due to the formation of ECM and collagen this phase is also called granulation phase. This name comes from the granular microscopic and macroscopic pattern of the tissue. A change in the fibroblast phenotype regulates inflammation, ECM formation, and angiogenesis during wound healing [11].

The final phase of wound healing process includes a remodeling phase lasting for months and years, starting a few weeks after injury [20]. The key event in this phase includes differentiation from fibroblasts to myofibroblasts [21]. The latter produces an ECM leading to formation of a mature scar (22). Myofibroblasts are nothing but modified dermal fibroblasts activated by various growth factors such as TGF-β1, TGF-β2 and TGF-β3 which appear in the wound area around 4 days after the injury [23–25]. The role of endothelial cells in the wound healing process is reflected in the formation of new blood vessels, which is called angiogenesis. This is important for providing oxygen supply and nutrients for tissue repair following the injury. The process of new blood vessels formation is based on the activation of locally present endothelial cells (ECs). The initial stimulus for EC activation is tissue hypoxia resulting from tissue damage. The next step involves the production of hypoxia-sensitive growth factors such as vascular endothelial growth factor (VEGF) or PDGF. VEGF-A receptors enable a higher level of control of this process. These receptors are expressed on the surface of endothelial cells and thus orient their migration towards the highest concentration of growth factors thereby forming a new vascular bed [14]. For the sufficient closure of the skin wound, it is necessary that along with the contraction of the wound, adequate re-epithelialization takes place, i.e., covering the wound with a new layer of keratinocytes. This includes migration, proliferation, and differentiation of keratinocytes in the wounding area [26]. Growth factors that support the migration of keratinocytes are epidermal growth factor (EGF) and transforming growth factor alpha (TGFα) which exert their effect through the same receptor on keratinocytes, called the EGF receptor [27, 28]. The initial mechanism for inducing the migration of basal keratinocytes in the direction of re-epithelialization is acute hypoxia caused by tissue injury due to clotting of dermal vasculature [29]. The transformation of keratinocytes from the so-called cobblestone-shaped cells into the flat migratory keratinocytes is considered one of the key events in this phase [14]. This process starts several hours after the injury. The first keratinocytes cover the injury site by the actions of so called lamellopodial crawling and shuffling [30, 31]. The wound is finally closed by the leading row of migrating keratinocytes covering the blood clot and the deposited extracellular matrix formed in the proliferation phase. The migration process will continue until the contact inhibition of keratinocyte in the wound center appears.

### Wound healing after traumatic brain injury

**Figure 2:**
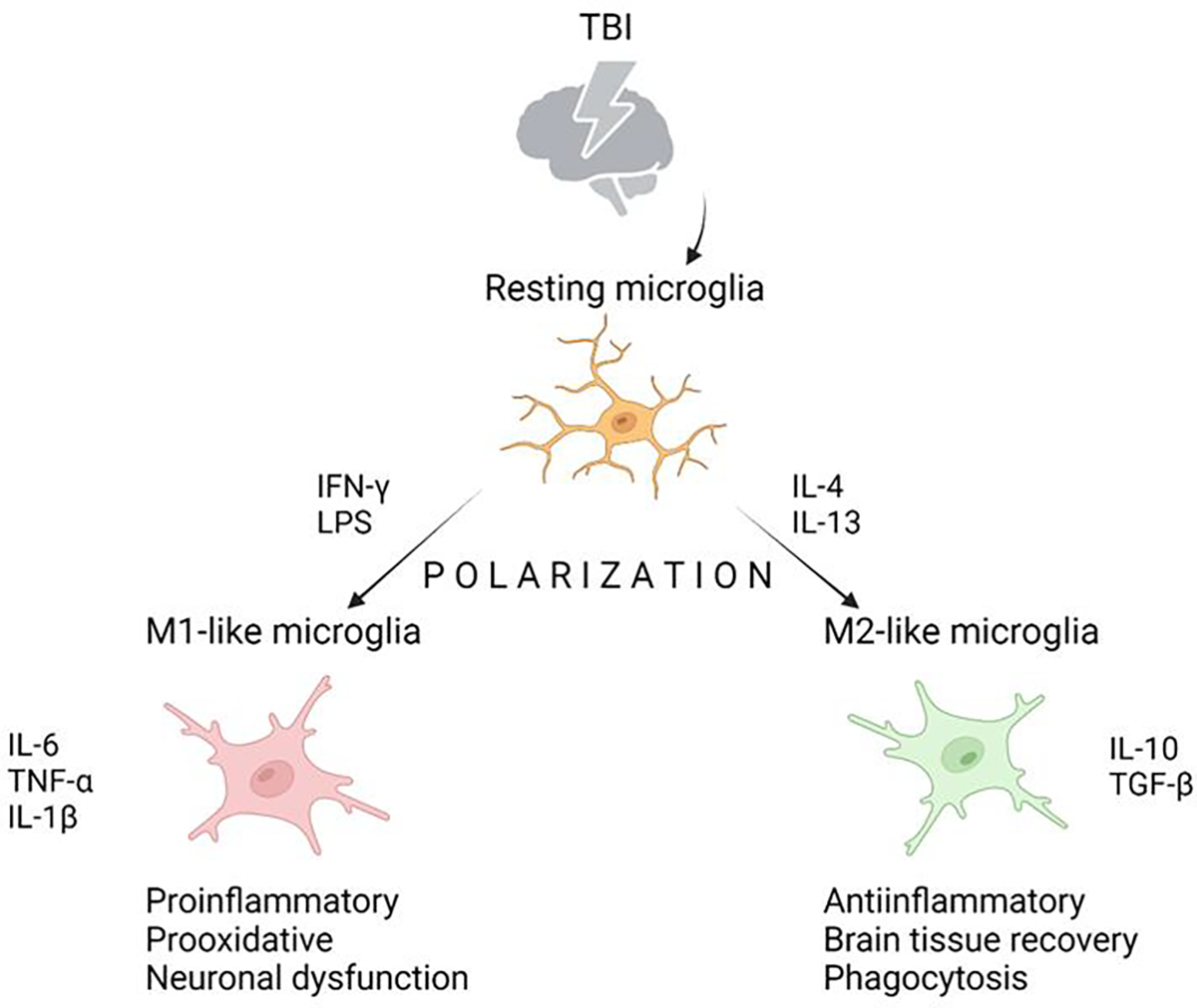
Microglia in response to traumatic brain injury (TBI). After TBI, in the interplay between damaged neurons and resting microglia, potentially two types of activated microglia, so-called M1-like microglia and M2-like microglia, could be differentiated. Polarization depends on local mediators and microenvironment. In response to LPS and IFN-γ, the M1-like phenotype arises, which produces proinflammatory cytokines and mediates neural dysfunction. M2-like phenotype is associated with tissue repair by producing anti-inflammatory cytokines and phagocytosis.

Traumatic brain injury (TBI) is defined as a brain damage caused by the action of an external mechanical force, which may result in a different degree of cognitive impairment or physical disabilities, and thus represents a significant public health issue [32, 33]. Approximately 30% of injury-related deaths in the US are due to the TBI [34]. For didactic reasons, TBI related tissue impairment have been divided into a primary (caused by the inertial or contact mechanical forces) and secondary injuries (due to numerous cellular dysfunctions, such as edema, inflammation, excitotoxicity, oxidative stress, or mitochondrial dysfunction) [33, 35]. Recovery from a TBI depends on many factors such as the localization or severity of the brain injury. Herein it is worth mentioning that the role of neuronal cells in tissue recovery following TBI is quite controversial due to its limited regenerative capacity. However, studies investigating different experimental models of TBI reported higher levels of neural stem cells activity following the injury [36]. This is primarily related to increased neural cell proliferation in the region of the hippocampus (dentate gyrus) and the subventricular zone [37–39]. Additionally, neurogenesis following TBI is associated with functional recovery of cognitive functions [40]. These findings may have a potential therapeutic implication in the treatment of the TBI consequences using stem cell-based therapy. Recently, the contribution of exosomes in neural tissue repair has been discussed and contributes to brain tissue repair by modulating cell differentiation [41]. Microglia is found to be one of the releasing sources of exosomes, in the conditions of serotonin and ATP stimulation [41]. In addition, it has been shown that phagocytosis by microglia plays a pivotal role in the process of recovery from traumatic brain injury, especially from the aspect of axonal regeneration [42]. Two microglial phenotypes were identified in response to brain damage, and in dependence on the microenvironment, the so-called M1 and M2 microglia (Figure 2). M2 phenotype is particularly important for inflammation reduction and promotion of brain tissue recovery following the injury. M2 polarization occurs mainly in the presence of IL-4 or IL-13 released by “resting microglia“ in response to mediators liberated from injured neurons [43]. Factors released from M2 cells that exhibit reparative properties are IL-10 and TGF-β [44]. A detailed analysis of reactive microgliosis following TBI is one of the potential steps for improving therapeutic strategies after TBI by modulating the inflammatory response.

### Effects of EMF on keratinocytes

**Figure 3:**
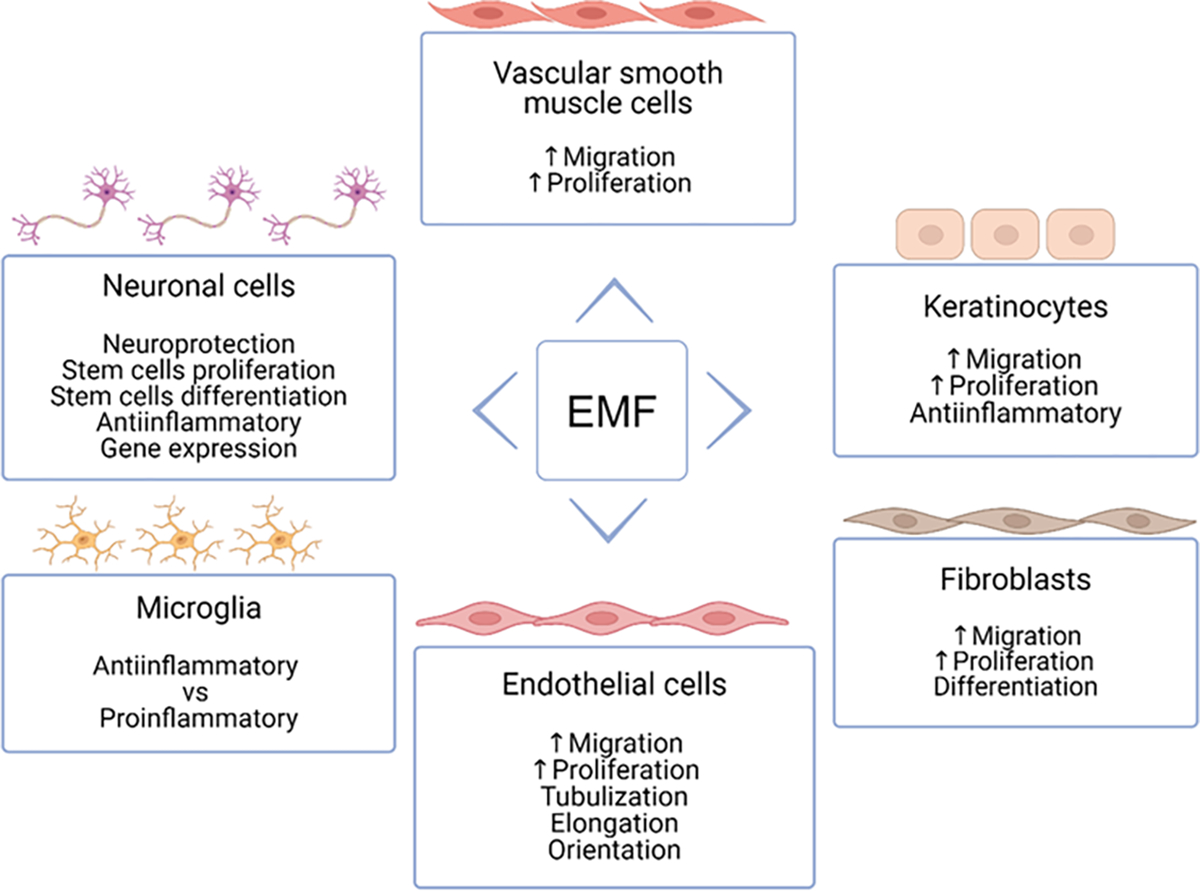
EMF effects on different cell types in terms of wound healing and TBI. Increased migration and proliferation of fibroblasts and keratinocytes indicate improved wound healing through the processes of ECM formation and epithelialization. Tubulization and elongation of endothelial cells in response to EMF speaks in favor of angiogenesis, promoting wound healing. In response to EMF neuronal stem cells proliferate, while microglia exert proinflammatory or anti-inflammatory response, depending on the study.

Keratinocytes play a critical role in wound healing by keratinocyte-fibroblast interaction and epithelialization. Decreased proliferation and migration of keratinocytes results in impaired wound healing [45, 46]. Application of EMF on keratinocytes reveals promising data in terms of improving re-epithelialization in the wound healing process via promotion of keratinocyte migration and proliferation (Figure 3). An *in vitro* study with human skin keratinocytes showed strong effects of EMF on stimulation of keratinocyte migration and a weaker effect on keratinocyte proliferation [47]. This study was conducted using EMF stimulation with a direct current (DC) for 90% of the time and reverse voltage for 10% of the time. The first EMF stimulation was performed 1 hour immediately after wounding and every 24 hours after for 4 consecutive days. For stimulation purposes, two frequencies of 980 Hz and 2080 Hz were used. The migration assay showed a statistically significant faster filling of the gap in the group treated with high frequency (2080 Hz) compared to the group treated at 980 Hz, but also compared to the control group without any EMF treatment. Additionally, EMF frequency stimulation of 2080 Hz led to complete filling of the wound three days earlier compared to the group treated at 980 Hz and the control group [47]. Therefore, according to this study, high frequency EMF and applied 1 hour and next 4 days after the wounding significantly stimulate keratinocyte migration. The proliferation assay performed in this experiment showed only slightly increase of keratinocyte proliferation.

Taken in more detail, the mechanisms involved in the effects of EMF stimulation are so far under-researched and controversial. Research conducted on HaCaT keratinocytes cell line showed that extremely low-frequency electromagnetic field (ELF-EMF) treatment (50 Hz, 1 mT) improves wound healing in two potential mechanisms: by accelerating early expression of IL-1β and by MMP-9 production. In this study, wound healing was assessed by measuring the cell free area at two hours intervals, starting from the initial scratching (T0) (48). Statistically significant decrease in cell free area was detected 8 hours and 24 hours after the initial scratching, meaning that ELF-EMF stimulation promotes the migration of keratinocytes [48]. Another study also applied ELF-EMF stimulation of 50 Hz at a flux density of 1 mT in different time intervals on HaCaT keratinocyte cell line aimed to determine its effect on cell proliferation and the production of different cytokines. The results showed a statistically significant growth rate of keratinocytes after 48 hours of ELF-EMF exposure in comparison to control nonexposed cells. Additionally, after 1 h of EMF stimulation NF-κB levels were almost undetectable, which has been suggested to reduce the production of different cytokines and chemokines such as: IL-8, monocyte chemoattractant protein-1 (MCP-1), macrophage inflammatory protein-1 α (MIP-1α) and regulated upon Activation, Normal T cell Expressed and Secreted (RANTES). The results of decreased production of proinflammatory cytokines following EMF stimulation suggest the use of ELF-EMF in the treatment of inflammatory lesions on the skin, especially in the wound healing process, if we consider the fact that persistent inflammation prolongs wound healing [49]. The role of EMF in the treatment of numerous musculoskeletal disorders such as bone fractures, osteoarthritis, osteoporosis and muscle and tendon injuries have been described [50]. For therapeutic purposes, the application of a special modality of EMF, the so-called radiofrequency EMF (RF-EMF), has been extensively investigated [51, 52]. However, the therapeutic use of RF-EMF as a method to accelerate skin wound healing has not been sufficiently elucidated so far. In a study, HaCaT keratinocyte cell lines were exposed to RF-EMF using a device that produces radiation with a frequency of 27.1 MHz (37 ns). To examine time dependence of the EMF stimulation three different protocols were used. In the first one EMF was applied two times 30 minutes each with 6 hours of non-exposure in between. The second and the third protocol included EMF continuously for 6 hours and 24 hours, respectively. The cells kept in the same conditions, but only without any EMF stimulation represented the sham-controls. The migration assay showed induction of keratinocyte migration following RF-EMF protocols. Additionally, RF-EMF contributes to wound closure in a timely manner with a significantly faster wound healing after 6 hours of EMF application with increased keratinocyte migration compared to untreated cells. Detection of gene expression after 6 hours of treatment showed statistically significantly higher levels of TGFβ compared to nonexposed cells [53]. This suggests that the induction of keratinocyte migration is mediated by the TGFβ related pathways. Additionally, the results showed that after 6 hours there was a significant increase of both the IL-6 gene expression and IL-6 concentration in cell supernatant, indicating that the early expression of IL-6 is of great importance for the rapid wound healing process [54].

### Effects of EMF on fibroblasts

Like keratinocytes, non-invasive EMF stimulation frequencies of 980 Hz and 2080 Hz did not produce significant effects on fibroblast migration and proliferation, compared to the untreated control [47]. However, application of extremely weak EMF (1 Hz) for 20 min on fibroblast showed significant increase in proliferation and migration fibroblasts. This was associated with a statistically significant increase in the expression of human fibroblast growth factor 1 (hFGF1) mRNA after EMF treatment, while expression of human vascular endothelial growth factor (hVEGF) was higher in the treated group but didn’t reach the statistical significance in comparison to control. The latter indicates activation of molecular wound healing pathways as a response to extremely weak EMF stimulation in an *in vitro* model of human fibroblasts suggesting therapeutic properties of EMF stimulation in wound healing [15]. An important aspect of wound healing, galvanotaxis or electrotaxis which is the ability of cells to migrate in response to electric field, is the endogenous electric currents that arise at the site of injury due to damage to the epithelial layer and consequential flow of positive ions from the surrounding tissue to the center of the wound. A study showed that stimulation of dermal fibroblasts with 100 mVmm^−1^ after one hour did not induce directional migration of fibroblasts, however, prolonged exposure of cells (up to 10 hours) with the same strength (100 mVmm^−1^), promote directional fibroblast migration towards the anode. Likewise, with the increase in the applied voltages above 100mVmm^−1^, there was a statistically significant increment in cell migration towards the anode. Western blot performed here showed a significant increase in the level of phosphorylated Akt (p-Akt) indicating PI3 kinase pathway as mediator of electrotaxis in an *in vitro* model of fibroblasts migration. These findings suggest that human dermal fibroblast migration is time and voltage dependent [55]. The beneficial role of ELF-EMF on fibroblasts is supported by an increase in human gingival fibroblast migration and proliferation after sinusoidal and pulsed ELF-EMF application with flux density of 1mT. Further, increased expression of proinflammatory cytokines including IL-6, TGF-β and MCP-1 after EMF application suggests the transition from the inflammatory to the proliferative phase of the wound reparation process and therefore promoting wound healing [56]. Contrary to the effects of ELF-EMF on fibroblast migration, Pasi et al. showed that ELF-EMF with frequencies of 5 Hz and 50 Hz, intensity in the range from 0.25 mT to 1.6 mT inhibits the proliferation of human dermal fibroblasts [57]. Decreased proliferation of fibroblasts was associated with an increased expression of tubulin, a component of the cytoskeleton responsible for the motility and shape of fibroblasts. Taken together, the combination of inhibited proliferation and increased tubulin expression refers to the transdifferentiation of fibroblasts to different phenotypes [57]. Previous reports also showed that the ELF-EMF exposure of 20 Hz and 7–8 mT affect the process of proliferation and differentiation of fibroblasts by modulating cyclic AMP-dependent protein kinase A (PKA) [58, 59]. In the light of recent research, new potential therapeutic modalities are emerging for the clinical application of EMF stimulation. In one experimental study Bioelectric Field Enhancement (BEFE) device was used to stimulate murine dermal fibroblast kept in media reconstituted with water solution. The specificity of this protocol is reflected in the fact that DC current in this research setting was applied through an aqueous solution, unlike most DC and AC experimental protocols [60] (Table 1).

### Effects of EMF on ECs and VSMCs

Adequate migration and proliferation of ECs is necessary for angiogenesis, one of the key events in the process of tissue regeneration during wound healing [10, 70]. Endothelial cells promote the process of angiogenesis by influencing the surrounding cells through the production of paracrine mediators [71]. Pulsed electromagnetic field (PEMF) promotes proliferation and tubulization of human umbilical vein endothelial cells (HUVECs). PEMF has clinical applications, and its mechanisms of actions promoting angiogenesis remain unclear. Exposure of endothelial cells to PEMF induces production of fibroblast growth factor β−2 (FGF-β2), and some other vascular growth factors such as angiopoietin-2 (Ang-2), thrombopoietin (TPO) and epidermal growth factor (EGF) suggesting its role in promoting angiogenesis [71]. ELF-EMF stimulation with intensity 1 mT, frequency 50 Hz, duration of exposure up to 12hrs (1h, 6h and 12h) to HUVEC cells showed a significant increase in cell proliferation and tubule formation suggesting its role in improving angiogenesis during wound healing. Furthermore, proliferation was increased in a timely dependent manner, showing a significant increase after 6 hours of EMF stimulation, in contrast to untreated controls. Results showed the highest migration rate after 1 h of exposure, even though in EMF treated groups the migration was increased in statistically significant manner, compared to control, at each time point (1h, 6h, 12h). It is well known that when plated on Matrigel coated plates, endothelial cells (HUVEC) make capillary-like formations (tube-formation assay) [72] and EMF application significantly increased tube formation compared to control. Another important finding was that reorganization of actin fibers nearby cellular membrane following EMF exposure, indicating participation of actin fibers rearrangement during improved wound healing after EMF exposure. EMF also affects wound healing via interplay between EMF and VEGF signaling pathway [73]. Since angiogenesis takes place mainly at the microvasculature level, and the process of new blood vessels formation influences the other vascular cells as well, Bai et al. [67] examined the effects of DC electrical stimulation of 150 to 400 mVmm^−1^ on the human microvascular endothelial cells (HMEC-1s), HUVECs, bovine pulmonary artery fibroblasts (BPAFs) and murine aorta smooth muscle cells (MASMCs). The results showed the influence of DC electric field (EF) on all four types of cells in terms of migration, orientation, and elongation. Migration of HMEC-1was fastest and directed toward the cathode, contrary to the other three cell lines where the migration was oriented towards the anode. In the light of these results, and bearing in mind that the processes of migration, elongation and cell orientation changes are involved in the process of vascular remodeling and angiogenesis, the potential use of EF stimulation in order to modulate angiogenesis can be considered [67]. However, more research is warranted to investigate the parameters for each cell involved in angiogenesis and wound healing.

Aimed to analyze the effects of LF-EMF on angiogenesis two types of stimulations were applied in both human (HUVECs) and mouse (MS1) endothelial cells: pulsed EMF (rectangular pulses, peak intensity 4 mT, frequency 72Hz) and sinusoidal EMF (peak intensity 6 mT, linear frequency increase from 1Hz to 100Hz followed by a 6 s delayed linear drop from 100Hz to 1Hz). The results revealed that pulsed EMF induce significant increase in human and mouse cells proliferation in comparison to both sinusoidal EMF treated group and control [74]. Another study examined the effects of LF-EMF on HUVECs and MS-1, similar to previous study, with EMF parameters including sinusoidal field, with frequency of 50 Hz and intensity of 2mT. The results showed significant inhibition of proliferation of both HUVEC and MS-1 mediated via VEGF pathway evidenced by western blot and immunofluorescence analysis [75]. Vascular smooth muscle cells (VSMCs), the most numerous cell population within the vessel wall, is located in the tunica media. Physiologically, VSMCs pertain contractile activity, however, in some pathological conditions VSMCs may acquire synthetic properties secreting a large amount of collagen, elastin and MMPs contributing to ECM remodeling and increases vasculogenesis following injury. In addition to differentiation to secreting cells, in response to tissue damage VSMCs also exert migration and proliferation properties [76]. VSMCs play a critical role in wound healing, however, the effects of EMG stimulation on VSMCs in respect to wound healing are quite scarce. One study investigated exposure of neonatal rat aortic smooth muscle cells (NRSMCs) to ELF-EMF for a constant period of 2 weeks. Sinusoidal stimulation was applied to the seeded cultures, with a frequency of 2 Hz and amplitude of 1.25 V/m. Electron microscopy analysis performed showed increased density of mitochondria, indicating enhanced mitochondrial production. The study also found that ELF-EMF exposure decrease elastin secretion without any changes in the composition of the elastin in terms of amino acids sequence compared to control [77]. The effects of low-energy EMF (36μT) on two types of vascular smooth muscle cells: bovine coronary SMC (bov-cSMC) and murine aortic smooth muscle cells (ms-aSMC) were investigated. Three frequences were applied (25 Hz, 50 Hz and 100 Hz) at 5 min, 15 min and 30 min of exposure. The results showed an increased number of cells induced by EMF for all frequencies tested, with the maximum number reached at frequency of 50 Hz. A significant increase in cell proliferation was observed in bov-cSMC exposed to EMF in two-fold manner, two exposures lasting for 15 min each, in 24 h intervals (with 50 Hz frequency) [78]. However, the mechanisms underlying the EMF effects on VSMCs are yet to be elucidated, as well as the parameters of application to promote wound healing and tissue regeneration in therapeutic clinical setting. A current study examining the galvanotactic response of ECs (HUVECs) and human umbilical artery smooth muscle cells (HUASMCs) exposed to DC electric fields reported that DC induces the migration of ECs and SMCs considerably, affecting the direction of migration as well as the level of migration [69]. The potential usage of galvanotaxis in clinical practice as a method of modulation of EC and SMC migration to promote wound healing and injury repair is yet to be studied.

### Effects of EMF on neuronal cells

There is a lack of published literature examining EMF effects on the neuronal cells, in-vitro. So far studies conducted on mice animal models showed anti-inflammatory and regenerative properties of EMF stimulation [79]. Additionally, in spinal cord transected rats EMF has been found to have some neuroprotective properties by affecting motor neuron excitability [80]. Some studies investigated the effects of EMF on neuronal stem cells and reported increased proliferation [81] and differentiation [82–84]. EMF has been found to have promising therapeutical properties in the treatment of TBI [85]. Despite the promising results in animal models, the translation of EMF in clinics remains equivocal, there is a need forfurther research on large animal models. Recently, we developed a experimental model of TBI by controlled cortical impact followed by EMF stimulation via a helmet on Yucatan miniswine to assess the effects on brain injury and repair that can better correlate to humans [86–88]. Our results in animal model receiving EMF immediately (20 minutes) after the TBI and two days after showed improved tissue repair in both swine but more histological improvement in terms of neuronal recovery and molecular mediators of inflammation in swine receiving EMF just after injury. The results of this pilot study suggests EMF as a potential therapeutic strategy to attenuate inflammation at injury site andimprove tissue recovery [12]. Since a small number of animals were used in this experiment, future studies are needed with increased number of animals to examine in more detail the potential protective properties of EMF stimulation on structural and functional changes after TBI.

Our group also reported [13] differential gene expression after TBI and EMF stimulation. There were several significantly differentially expressed genes in this study. These include Inscuteable Spindle orientation Adaptor Protein (INSC), Transthyretin (TTR), Cilia and Flagella Associated Protein 126 (CFAP126), Semaphorin 3F (SEMA3F), Calbindin 1 (CALB1), Cadherin 19 (CDH19) and Serine Proteinase Inhibitor (serpin) Family E member 1 (SERPINE1) (13). These genes regulate wide array of cellular functions such as proliferation, migration, thyroid hormone transport, reactive oxygen species regulation, or immune cell infiltration, and inflammation. The change in gene expression due to EMF exposure was time-dependent after TBI. Further, the differentially expressed genes (DEG) such as Adenosin G Protein Coupled receptor G3 (ADGRG3), Adenosin G Protein Coupled receptor G5 (ADGRG5), Phospholipid-transporting ATPase 8B3 (ATP8B3), Lipase G (LIPG), Cytochrome P450 Family 19 Subfamily A Member 1 (CYP19A1) and Leucine Rich Repeat Containing 2 (LRRC2) were significantly upregulated in the injured compared to non-injured area [89]. These findings indicate the potential usage of EMF stimulation as a method to improve recovery after TBI due to its capacity to alter gene expression regulating various cell functions. However, future studies should be performed to better explain the EMF effects on gene expression under different stimulation parameters to delineate the best suitable parameters and improve EMF efficacy.

### Effects of EMF on microglia

Microglial cells, a part of innate immune system of the brain have phagocytotic properties, act as central nervous system macrophages [90]. In communication with other cells in the microenvironment, such as neurons and astrocytes, they maintain the homeostasis of the brain tissue [91]. Their function is particularly pronounced in various pathological conditions of the central nervous system such as ischemia, hemorrhage, or trauma. Microglial cells change their morphology with a change in gene expression due to increased secretion of various cytokines and growth factors [92]. Microglia exert both beneficial and detrimental roles in response to brain trauma because of their contribution to tissue recovery in the initial stages while their prolonged activation leads to chronic inflammatory response [44, 93, 94]. Considering the significant impact of microglial cells on post-injury inflammatory response, the effects of PEMF stimulation were investigated on microglia treated with different LPS concentrations. The N9 murine microglial cell lines were used in this study, since they have been proven as a good model to examine the inflammatory response [95]. PEMF stimulation was applied with the following parameters: 75 Hz frequency, 1.3 ms pulse duration, 1.5–0.2mT the peak intensity of the magnetic field. The study concluded that treatment with LPS concentrations ranging from 0.1 to 1 μg/ml activated the N9 microglial cells leading to significantly increased production of TNF-α, IL-1β, IL-6, and IL-8 and PEMF exposure significantly reduce the production of the cytokines. PEMF was shown to exert direct anti-inflammatory propertiesand reduced the hypoxia-induced ROS production in the microglia cell [96]. Since the proinflammatory cascade is activated in a response to ischemic pathologies, the clinical usage of PEMF as a potential therapeutic option for the treatment of stroke conditions has been suggested. Considering that TBI is partially an ischemic injury, the PEMF exposure following TBI could also be suggested as a therapeutic option. Interestingly, the results of this study [96] were opposite to the study mentioned above [95]. The increased TNF-α and iNOS mRNA expression in microglial cells measured 1h following the 2.45 Hz EMF exposure lasted for 20 minutes may be due to the activation of JAK2-STAT3 signaling pathwayafter EMF application [97]. It has been reported that this signaling pathway contributes to the proinflammatory effects of EMF exposure on microglial cells but does not participate in the early stages of microglial activation [98]. These findings suggest that inhibition of JAK2-STAT3 signaling could be considered as a therapeutic option for prevention of EMF induced microglial activation in case of persistent inflammation.

## Conclusion

EMF stimulation significantly affects migration and proliferation of different cells involved in crucial processes of wound healing such as angiogenesis, ECM remodeling, or epithelialization and tissue repair after injury. However, there is a paucity of evidence to translate the LF-EMF to the clinics to enhance wound healing such as in chronic ulcers and diabetic foot ulcers and tissue and neuronal repair after traumatic brain injury or spinal cord injury. The incomparable results of various reports involving different stimulation parameters, exposure times, and cell types warrants future research to define the optimal parameters. Testing different stimulation frequencies to delineate the most effective frequency and the time and duration of exposure with a particular frequency to translate and improve clinical outcomes is the need of time.

## Figures and Tables

**Table 1:** Overview of the studies that reported effects on cellular migration/proliferation in respect to applied AC or DC currents.

Cell type	Field type	Migration	Proliferation	Reference
Fibroblasts, human	AC	Stimulation	Stimulation	56
Keratinocyte, human	DC	Stimulation	Stimulation	47
3T3 Fibroblasts, rat	DC	Stimulation	ND	61
3T3 Fibroblasts, rat	DC	Stimulation	ND	62
Keratinocytes, human	DC	Stimulation	ND	63, 64
Vascular endothelial, human	DC	Stimulation	ND	65
Aortic endothelial, bovine	DC	Stimulation	ND	66
Fibroblasts, murine	DC	Stimulation	ND	60
HMEC-1; BPAF; MASMC; HUVEC	DC	Stimulation	ND	67
Fibroblasts, Keratinocytes, murine	DC	Stimulation	ND	68
HUVECs, HUASMCs	DC	Stimulation	ND	69
HMEC-1, human microvascular endothelial cells; BPAF, bovine pulmonary artery fibroblasts; MASMC, murine aorta smooth muscle cells; HUVEC, human umbilical vein endothelial cells; HUASMCs, human umbilical artery smooth muscle cells; ND - not determined.				

## Key points, Outstanding Questions and Challenges

- EMF stimulation increases the proliferation and migration of cells involved in the physiological process of wound healing, such as fibroblasts, keratinocytes, vascular smooth muscle cells and endothelial cells.
- Recent studies on large animal models reported the protective effects of EMF on the post-TBI recovery in terms of reducing inflammation and affecting differential gene expression.
- Scientific evidence indicates EMF application in clinical practice as a potential therapeutical treatment aimed to accelerate both the tissue repare in the wound healing process e.g. diabetic foot ulcer, or post-TBI tissue recovery.
- What are the optimal EMF stimulation parameters for the effective clinical implementation?
- Which mechanisms underlie the effects of EMF on the migration and proliferation of cells involved in wound healing, and on proliferation and differentiation of cells in the process of recovery after TBI?
- One of the future challenges in this field is to establish an EMF exposure protocol with the most effective stimulation parameters (exposure time and frequencies) obtained from in vitro analysis, so it could be applied in an in vivo experimental model, and finally translated to clinical usage in human population.
